# Disclosure of medical errors to patients in China

**DOI:** 10.2471/BLT.14.149765

**Published:** 2015-05-15

**Authors:** Lei Gao, Xuhong Zuo, Han Xu, Xiang Gao, Yanguo Chen, Chun Cecilia Wang, Shaojun Wang

**Affiliations:** a Department of Ophthalmology, Yantai Yuhuangding Hospital, Shandong, China.; b Fuwai Cardiovascular Medical Center, Beijing, China.; c Department of Ophthalmology, Weifang Eye Hospital, 199 Xingfu Road, Weifang, China.; d Department of Ophthalmology, Laiyang Chinese Medicine Hospital, China.; e University of Chicago Law School, Chicago, United States of America (USA).; f Cardiology Division, Kaiser Permanente Richmond Medical Center, 901Nevin Ave, Richmond, CA 94801, USA.

Disclosure of medical errors or other adverse events to patients is handled differently in China compared to many other countries.^1^^–^^3^ Many doctors in China feel uncomfortable with discussing complications or errors with patients and few recognize an ethical obligation to disclose errors.^1^^,^^4^ The standard approach to such incidents is to say nothing to the patient unless there are serious long-term consequences. Here we share our experience of a patient who suffered complications during eye surgery and how the surgeons handled the case. We suggest improvements to the standard approach used to handle medical errors in China.

The patient was a 76-year-old man with long-term visual impairment. He had lost vision in his left eye 12 years previously and was scheduled to have an operation on his right eye for retinal detachment. Most intraocular operations are performed under local anaesthesia worldwide. It has been suggested that these local anaesthetic procedures may not be as safe as initially thought.^5^

The patient consented to the procedure. After local anaesthesia was administered, the surgeon immediately discovered acute vitreous haemorrhage and a small retinal laceration consistent with needle injury. He proceeded quickly and completed the procedure without any other difficulties. Three months later, after several additional operations, the patient had recovered his visual acuity in the right eye to 20/60.

The iatrogenic retinal injury was never disclosed to the patient. For a long time, the surgeon was uneasy about not disclosing the complication, particularly when the patient repeatedly expressed his gratitude for the eventual favourable outcome. The patient had no knowledge that a serious complication had occurred. Several months after the incident, the surgeon presented this case at a national meeting of ophthalmologists. His peers acknowledged the ethical problems at hand, but responded that in the context of deteriorating conditions of medical practice in China, the complication should not be disclosed, as there were no permanent medical consequences for the patient or financial consequences for the surgeon.

In many countries, informed consent is obtained before surgery.^3^^,^^6^ Discussion covers the nature of the procedure, possible complications and alternative procedures, so that patients can make informed decisions. After operating, surgeons are required to fully disclose the outcomes of the surgery and any complications. When complications do occur, they should be discussed to identify modifiable causes, whether individual or systemic.^3^

China has over 3000 years of medical and surgical practice. Historically, doctors were the sole decision-makers, trusted to act in the best interests of each patient. They were regarded as scholars with the highest standards of knowledge and ethics.^7^ Unfortunately, this tradition of trust has gradually deteriorated over time, especially during the last three decades of rapid economic development. Trust has been eroded as both patients and physicians have acted for personal gain in some cases.^1^^,^^4^ Legislation on patient rights has not kept pace with these problems. Regulations are scattered in ambiguous and poorly-enforced civil codes;^8^^–^^10^ arbitration or other legal procedures to solve disputes are poorly defined and not standardized.^9^^,^^10^

In 2010, tort liability legislation came into force in China.^11^ This law emphasizes the patient’s right to information and the need for doctors to discuss potential complications and alternative treatments. Since the implementation of the new law, there has been some progress in improving informed consent procedures before operations, but disclosure of errors is still at the provider’s discretion.^4^

We have identified several problems with the current situation. First, while the new law is a step forward, it lacks details and clear definitions, and the recommendations on solving disputes are vague and nonspecific. In China, most disputes are handled by arbitration organized by local health-care officials, often viewed by patients to be biased.^1^^,^^4^

Second, medical ethics’ education is very limited and physicians remain strongly influenced by a paternalistic tradition.^1^^,^^4^^,^^7^ They are not acquainted with basic ethical principles of autonomy, beneficence, non-maleficence and justice. Their decision to disclose errors is often based on eventual clinical or financial impact, rather than out of respect for patient autonomy or basic rights.^7^

Third, most hospitals in China are for-profit institutes;^4^^,^^7^ physicians are paid based on productivity. There is concern that full disclosure of alternative treatment would cause patients to withdraw from procedures. Finally, it is not uncommon to see cases where the effects of complications are exaggerated by patients. This has led to unnecessary sanctions imposed on doctors and unfair financial rewards to patients.^1^^,^^4^

Ethical regulations in China,^8^^,^^10^^,^^11^ the way doctors are trained, the need to avoid baseless accusations of malpractice^4^ and the current profit-based health-care delivery model^1^ have all contributed to this dilemma. The core of the problem is a lack of clarity and poor enforcement of existing legislation. As the cause of these challenges dates back thousands of years, the problems are unlikely to be solved quickly.

We believe the following steps need to be taken to change the current ethical standards and to reverse the deterioration in patient–doctor relationships. First, we urge the state legislators to review international ethical standards and apply these to China. Clear definitions are needed for terms in civil codes and ambiguous words such as “probable” or “may be,” need to be removed. Second, ethical education in medical schools and training facilities is needed. Third, the medical community should start a discussion about the use of medical malpractice insurance to protect both doctors and patients. Fourth, institutional policies should be designed in the interests of patients, rather than being profit-driven. To protect doctors from wrongful accusations, limits to doctors’ liability for common procedural complications should be established. Lastly, the ethical and technical performance of clinicians should be regularly monitored and reviewed.
